# Overlapping Features of Psoriasis and Atopic Dermatitis: From Genetics to Immunopathogenesis to Phenotypes

**DOI:** 10.3390/ijms23105518

**Published:** 2022-05-15

**Authors:** Ya-Chu Tsai, Tsen-Fang Tsai

**Affiliations:** 1 Department of Dermatology, Far Eastern Memorial Hospital, New Taipei 220, Taiwan; gracybamboo@gmail.com; 2 Department of Dermatology, National Taiwan University Hospital and National Taiwan University College of Medicine, Taipei 100, Taiwan

**Keywords:** atopic dermatitis, psoriasis, overlap, concomitant, paradoxical, psoriasis dermatitis

## Abstract

Psoriasis (PSO) and atopic dermatitis (AD) were once considered to be mutually exclusive diseases, but gradually regarded as a spectrum of disease. Shared genetic loci of both diseases were noted in some populations, including Chinese. Shared immunopathogenesis involving Th17, Th1, Th22 cells, or even IL-13 was found in certain stages or phenotypes. This review discusses the overlapping genetic susceptibility, shared cytokines, immune-mediated comorbidities, and clinical presentations. Overlapping conditions could be classified into mainly PSO lesions with AD features or vice versa, concomitant PSO and AD, or disease transformation as a result of biologics treatment.

## 1. Introduction

Psoriasis (PSO) and atopic dermatitis (AD) are common inflammatory skin diseases with distinct clinical manifestations [1]. The prevalence of PSO is 2% in Caucasians and 0.2% in Asian regions [2,3]. AD has a much higher prevalence, especially in children (up to 20%) [4,5]. PSO and AD each have their own typical involvement areas. PSO tends to occur on the scalp and extensor skin, while AD varies with age, i.e., the extensor side of extremities and face in infancy; flexure side and hands in adolescents and adults.

Historically, opposing immunopathogenic mechanisms, Th2 and Th1, had been proposed for these diseases. Besides, different RNA transcriptomes [6] and barrier profiles have been revealed [7]. With regard to the disease course, PSO has a peak onset around 20–30 years of age, and most patients continue to suffer throughout life, while AD usually begins in early childhood with improvement before adolescence. In fact, patients with PSO were reported to have a 25-fold lower prevalence of AD [8]. Thus, the concurrence of PSO and AD was once considered to be very rare.

**Figure 1:**
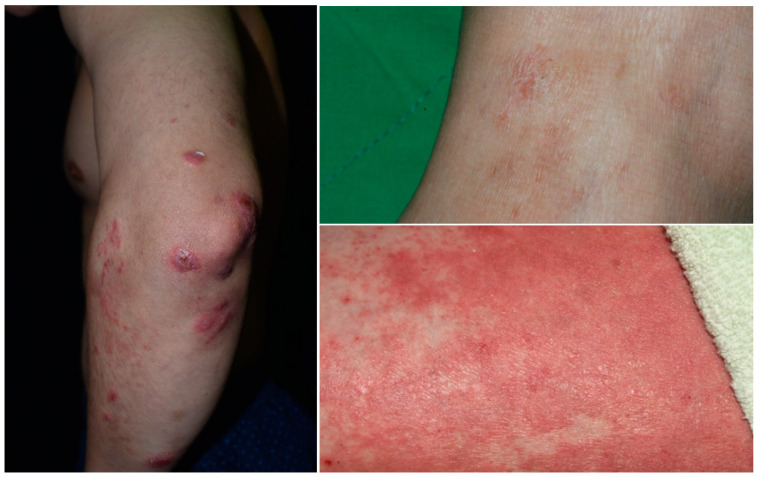
Psoriasis with overlapping features of eczema.

However, a recent study has shown a directional association between PSO and AD [9]. There are shared genetic profiles, immune pathways, pathologic changes, and comorbidities for the diseases. Indeed, retrospective and case studies about concomitant AD and PSO have been increasingly reported [10,11,12,13]. They may occur as diseases with overlapping features or coexisting diseases on different body regions in the same individual (Figure 1). Conventional oral immunosuppressive therapy and phototherapy can treat both AD and PSO, but biologic agents targeting only specific T-cells or cytokines are often ineffective for concurrent diseases, and might even induce transformation from one disease to the other. This review includes literature from PubMed and Google Scholar by searching “(shared, concomitant, coincident, overlap, coexist, concurrent) AND (psoriasis, atopic),” as well as pertinent references by manual searching.

## 2. Shared Genetic Background

Both PSO and AD have a strong genetic background, with family segregation and higher disease risks in monozygotic twins compared with dizygotic twins. Dozens of genetic loci have been discovered in the diseases, respectively, which correspond to the genetic *heterogeneity*. The most frequent susceptibility gene locus of PSO is HLA-Cw*0602 (on PSORS1 6p21), while null mutations of the FLG gene is the strongest genetic risk for developing AD [1,2,3,5].

In terms of genes on the overlapping chromosomal loci of the diseases, contrasting results were revealed in comparative studies. Most studies indicated that the epidermal differentiation complex (EDC) on chromosome **1q21.3** contains FLG gene mutations for AD, which has no relation to PSO [14,15,16]. Likewise, the late cornified envelope (LCE) genes 3B/3C deletion within the EDC for PSO is not related to AD [14,17]. However, variants of FLG mutation were reported to confer a risk of developing psoriasis in Taiwanese and Chinese populations [18,19].

Another shared region on the genome is chromosome **5q31.1-q33.1,** where IL-13 has shown associations with both AD and PSO [14]. Previous data indicated that IL-13 was a signature cytokine of AD, more important than IL-4 [20]. Baurecht et al. proposed the opposing risk alleles at this shared locus (chromosome 5q31.1, the Th2 cytokine control area) of the diseases [14]. However, there was also evidence to support the relationship between IL-13 and PSO/psoriatic arthritis [21,22]. In brief, most genetic analyses are in favor of AD and PSO as opposing diseases, but overlapping loci or shared cytokines have been noted, although their influence on diseases remains unclear.

## 3. Shared Immunopathogenesis

### 3.1. PSO

Genetic predisposition and environmental triggers interact to induce PSO. Stimulated keratinocytes release antimicrobial peptide LL37, which further amplifies toll-like receptor 9 signaling on plasmacytoid dendritic cells (pDC). Activated pDCs produce interferon (IFN)-α, which enhances myeloid dendritic cell (mDC) maturation. IFNα is also related to the differentiation of Th1 and Th17 and the production of IFNγ and IL-17. Myeloid DCs can be activated via LL37 as well. After activating, mDCs migrate to draining lymph nodes to release TNFα and IL-23. IL-23 modulates Th17 cell proliferation and maturation. Th17 secretes IL-17 and IL-22 combined with TNFα and IFNγ to induce keratinocyte hyperproliferation and undifferentiation. IL-17 is mainly secreted by Th17 and type 3 innate lymphoid cells (ILC3) in psoriasis. Overall, the TNFα-IL23-Th17-IL17A/F pathway is the hallmark of plaque-type psoriasis. On the other hand, pustular psoriasis involves the mutation of the IL-36 receptor antagonist secreted by keratinocytes [1,2,3].

### 3.2. AD

A combination of genetic background, epidermal barrier defects, microbiome imbalance, and immune dysregulation contribute to AD. Although the Th2 pathway is the main driving pathway of AD, the multipolar involvement of immune axes leads to various phenotypes and severities. The addition of environmental stress on epidermal barrier defects activates dendritic cells and type II cytokine-related response. Stimulated Th2 cells release IL-4, IL-13, IL-31, and keratinocytes produce IL-33 and TSLP, driving further inflammation and barrier dysfunction. Type 2 innate lymphoid cells (ILC2) generate IL-5 and IL-13 cytokines, which recruit eosinophils and Th2 cells. The accompanied elevation of IL-22, mainly produced by Th22 and Th17, inhibits keratinocyte differentiation and induces epidermal hyperplasia. Moreover, Th1 plays a role in chronic AD, while the Th17 axis relates to the Asian or pediatric types of AD. However, trials regarding the Th17 pathway failed to achieve adequate efficacy for moderate to severe AD, so its clinical significance still needs further evaluation in AD. In terms of atopic march (allergic march), it might be related to chronic IgE sensitization by IL-4 and IL-13 [1,4,5,23].

### 3.3. PSO and AD

**Figure 2:**
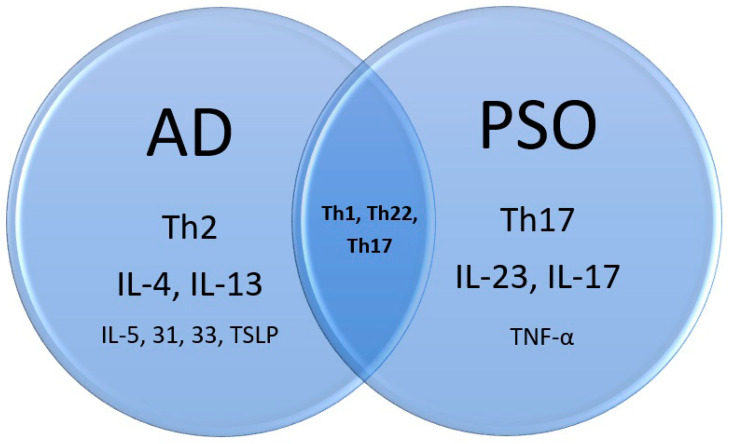
Immunopathogenesis of atopic dermatitis, psoriasis, and the overlap.

PSO is mainly an IL23-Th17-IL17 disease, while AD is Th2 skewing associated with IL-4 and IL-13. Nevertheless, Asian, pediatric, and intrinsic types of AD involve Th17 as well [1,23,24]. Analysis of PSO susceptibility genes identified an odds ratio of 1.18 increase in IL-4/IL-13 signaling loci [25]. Furthermore, both diseases involve Th1 and Th22. However, IL-22 might not be essential to PSO and AD, since the blocking of IL-22 did not prove its efficacy in PSO treatment [26] and was only moderately effective for AD in a phase 2a trial [27]. A following study in AD was suspended, accordingly. Although IL-22 levels were increased in both diseases, it might not be the culprit, but an innocent bystander (Figure 2).

IgE was often used clinically as a surrogate serum marker for atopic diathesis, including AD. Despite this, total IgE levels were significantly higher in psoriatic patients than in healthy controls (median IgE 425 IU/mL versus 54.5 IU/mL, *p* < 0.05) [28,29]. Additionally, patients with a longer period of psoriatic skin lesions had a statistically significant elevation of total IgE levels too [28]. Regarding the mite test measured by the prick test, subjects in PSO and asthmatic groups showed statistically significant positive rates compared to individuals in the healthy control group [29].

In vitro, the scratch injury from both diseases induces CCL20 production by keratinocytes. CCL20 then chemoattracts IL17-producing immune cells. This is another explanation for why the IL-17 amount is also increased in AD [30]. However, pruritus signaling is quite different in AD and PSO. Substance P, IL-2, calcitonin gene-related peptide (CGRP), OPRM, and OPRK are involved in psoriasis-related itch, while thymic stromal lymphopoietin (TSLP), CGRP, IL-4, IL-13, and IL-31 are associated with AD pruritus. Psoriasis itch is mainly induced by transient receptor potential vanilloid 1 (TRPV1) channel, but AD itch is mainly through transient receptor potential ankyrin 1 (TRPA1) [31,32,33].

## 4. Histopathological Findings

**Figure 3:**
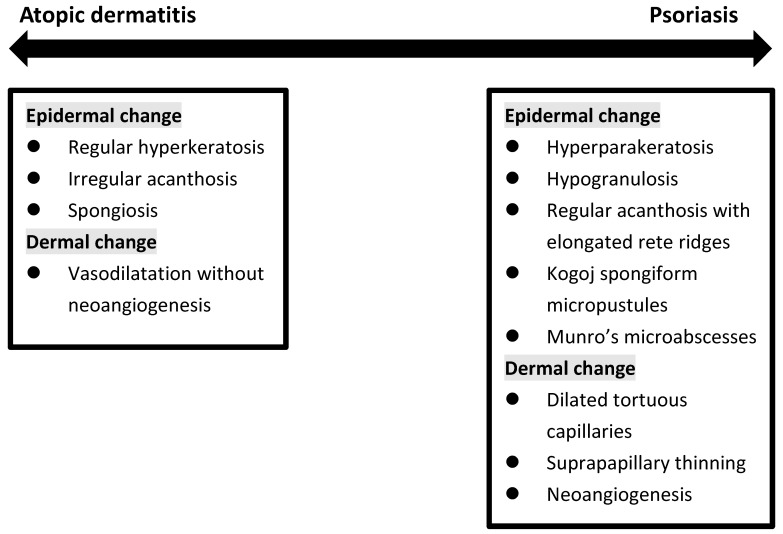
Histopathologic change in between atopic dermatitis and psoriasis.

Histopathologically, PSO is characterized by sparse superficial perivascular lymphocytic infiltrates and the extension of lymphocytes into the epidermis in the early phase. It is followed by retention of nuclei (parakeratosis) and mounds of neutrophils (Munro’s microabscesses) in the stratum corneum, elongation of epidermal rete ridges with characteristic bulbous enlargement of their tips or clubbing, i.e., psoriasiform hyperplasia and tortuous vascular ectasias in close proximity to the basal layer [34,35]. In contrast, the histopathological findings of AD are much less characteristic. Intercellular edema within the epidermis, namely spongiosis, is the hallmark of all dermatitis, including AD. The degree of spongiosis depends on the stage of lesions, with more vesiculation in the acute phase and irregular epidermal hyperplasia in the chronic phase (Figure 3).

The diagnosis of inflammatory skin diseases is heavily dependent on clinical signs [36]. However, clinicians sometimes face a dilemma when there are characteristics in between AD and PSO. It is especially problematic when irritation or partial treatment is accompanied by lesions on volar skin and in patients with erythroderma [37]. Pathology is hence the next step to make a further distinction. In actuality, atypical histopathologic features of PSO are noted with high probability because dermatologists rarely perform biopsies on skin lesions that show typical clinical features of PSO for diagnostic purposes. AD, especially in its chronic form and impetiginization, can share many similar histopathologic features with PSO.

Eosinophilic leukocytes were deemed absent in PSO [38], but are regularly observed in AD. In fact, in a case series of 51 clinically confirmed cases of PSO, spongiosis, compact orthokeratosis, dermal plasma cells, and dermal eosinophils were seen in 76%, 37%, 21%, and 49%, respectively. Spongiosis was 100% present in guttate PSO, and eosinophils were identified in 80% of inverse PSO. In palmoplantar PSO, dermal plasma cells were observed in 50% of patients [39]. In another two studies, eosinophils were seen in 46% [40] and 18% [41] of biopsy specimens of PSO. It is not uncommon to see a pathologic diagnosis of psoriasiform spongiotic dermatitis or spongiotic psoriasiform dermatitis, which turned out to be PSO or AD after follow-up.

Among patients with hand eczema, around one-third of moderate-to-severe hand diseases had a history of AD [42]. Non-pustular palmoplantar PSO has considerable clinicopathologic overlaps with hand eczema. In a cohort of 132 patients having palmar inflammation, a mixed histology of eczema and PSO was given by pathologists in 77 patients [43]. For palmoplantar lesions, although findings of psoriasiform hyperplasia, parakeratosis, hypogranulosis, presence of Munro’s microabscesses, and appearance of tortuous and ectatic capillaries in the papillary dermis were more frequently seen in palmoplantar PSO compared with eczematous dermatitis, none of these features were statistically significant. Conversely, spongiotic vesicles were noted in a high proportion of the patients with PSO (76.5%) [44]. A retrospective study revealed similar results: it failed to attain the histopathologic distinction between palmar PSO and hyperkeratotic hand eczema [45]. In one immunohistochemical analysis, hyperkeratotic hand eczema was found to share pathogenesis with palmar PSO, based on the elevated level of β-defensin 2 in the stratum corneum layer and IL-36γ in the stratum granulosum layer in both diseases [46].

## 5. Shared Comorbidities Focusing on Autoimmune Diseases

A meta-analysis demonstrated that multiple autoimmune diseases had a varying extent of association with AD. This included alopecia areata, vitiligo, celiac disease, ulcerative colitis, Crohn′s disease, rheumatoid arthritis, and systematic lupus erythematosus [47]. Genetic cause has been suggested because there is a greater risk of alopecia areata in patients with filaggrin gene mutation [48]. AD shared 39 genetic loci with inflammatory bowel diseases, which also implied a genetic linkage [48].

In PSO, the prevalence of alopecia areata, vitiligo, rheumatoid arthritis, systemic lupus erythematosus, bullous pemphigoid, and pemphigus were increased (Table 1) [49].

### 5.1. Alopecia Areata

Both PSO and AD have a higher risk of developing alopecia areata. Relative risk is 4.71 in patients with PSO [49], and the hazard ratio is 6.00 in patients with AD [50], using the same database. Conversely, among patients with alopecia areata, the risk of PSO and AD is also increased, but it differs with onset age. The odds ratio of developing AD is 3.82 in alopecia areata patients younger than 10 years old, while PSO risk appears in the group of onset age between 11 to 20 years of alopecia areata (odds ratio: 2.43) [51].

### 5.2. Vitiligo

The comorbidity profile of vitiligo includes PSO and AD [52]. The reverse is also true. The relative risk of developing vitiligo is 1.64 for AD compared with controls in two cohort studies [53,54]. Vitiligo also has an elevated incidence in patients with PSO (Relative risk: 5.94) [49].

### 5.3. Inflammatory Bowel Disease

Most studies revealed a higher incidence of inflammatory bowel disease in PSO, especially Crohn’s disease [55,56]. However, some cohort studies showed no significant relationship between PSO and ulcerative colitis. A connection between celiac disease and PSO was also observed in a number of studies [57,58]. Nevertheless, whether a gluten-free diet benefits PSO is still doubtful. As for AD, it increased the risk of Crohn’s disease, ulcerative colitis, and celiac disease [59,60,61].

### 5.4. Others

Rheumatoid arthritis has long been reported to be more common in patients with PSO [49,58,59]. However, less well known is that the association is also seen in patients with AD [60,62]. Likewise, asthma and allergic rhinitis are the well-recognized comorbidities of AD. However, asthma and allergic rhinitis are also increased in patients with PSO [49,59].

Bullous pemphigoid is characterized by subepidermal tense blisters with eosinophilic infiltrates. It is the most frequently reported autoimmune bullous diseases related to PSO [49,63]. Traditionally, it was found to be increased in patients with PSO only. More recently, an association with AD was also reported [64,65].

## 6. Phenotypes of Overlapping Psoriasis and Atopic Dermatitis

**Figure 4:**
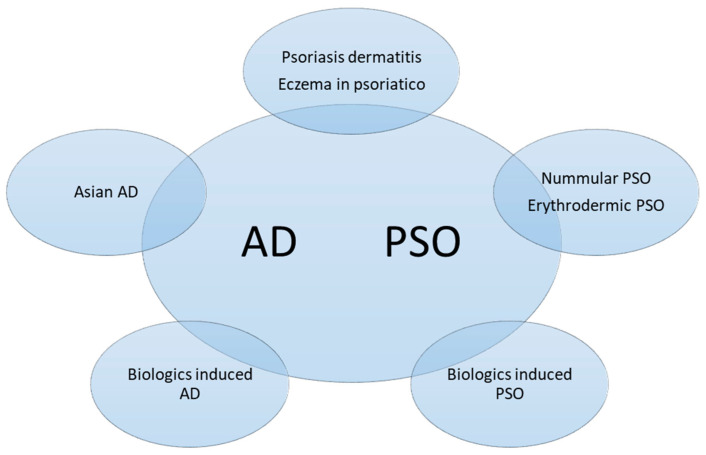
Subtypes of overlapping psoriasis (PSO) and atopic dermatitis (AD). The phenotypes could be classified into concomitant PSO and AD, mainly PSO lesions with AD features or vice versa, or disease transformation as a result of biologics treatment.

Although the diagnosis of typical PSO and AD is usually straightforward, it may be more challenging in the pediatric group or in special locations. In a study of pediatric PSO and AD, only 10% of children with PSO were diagnosed correctly, and 79.9% of patients with PSO were diagnosed as AD by the referring doctors [66]. Lack of experience might be one reason; lack of typical lesions would be another reason. Even dermatologists may sometimes find it difficult to make a clear distinction in 20% of cases that showed a combination of both disease features, so-called psoriasis eczema (PsEma) [67]. Overlapping diseases can be diagnosed concurrently or consecutively. In order to specify various conditions, we subdivided them into five subtypes according to the clinical manifestations, disease course, and transformation induced by medications (Figure 4):

1. PSO with AD features (Nummular PSO, erythrodermic PSO)
2. AD with PSO features (Asian AD)
3. Coexisting AD and PSO (Psoriasis dermatitis, PSO-Eczema, PsEma, eczema in psoriatico)
4. Development of AD-Like dermatitis during PSO or AD treatment (TNFai, IL-12/23i, IL-17i, IL-23i, IL-4/13i)
5. Development of PSO during AD treatment (Dupilumab)

### 6.1. PSO with AD Features (Nummular PSO, Erythrodermic PSO)

Nummular PSO is a variant form of plaque PSO. It mainly locates on four limbs with intense itch. Clinical features resemble nummular eczema. The size is usually 4 to 5 cm in width, and the shape is annular, so-called “nummular” [68]. Nummular PSO might be the initial sign of PSO during childhood [69].

Patients with erythrodermic PSO showed not only a Th1 response, but also an inclination toward the Th2 axis [70]. Biopsy specimens from erythrodermic PSO and AD found that the Th1: Th2 ratio had no significant difference. Besides, there was no significant difference in the percentage of CD3+T cells, including Th1, Th2, Th17, and Th22 cells. Thus, there might be an immunologic overlap of Th17 and Th22 between erythrodermic PSO and AD [71]. Serum IgE level of erythrodermic PSO was much higher than that of healthy control [72] and plaque-type PSO (81.3% versus 6.3%) [73]. Peripheral blood eosinophilia was noted in 41% of patients with erythrodermic PSO [74].

### 6.2. Asian-Type AD

Asian AD is a phenotype of AD with properties between European/American (EA)-AD and PSO. Epidermal changes of psoriasis, including acanthosis, Ki-67 levels, and parakeratosis, were all significantly increased in Asian AD versus EA-AD. Although typical AD demonstrated acanthosis as well, Asian AD showed more psoriasiform hyperplasia with hypogranulosis, elongation of rete ridge, and parakeratosis. Besides, neutrophil infiltration, another feature of psoriasis, was more often observed in Asian AD than in EA-AD. With respect to immunopathology, both Asian and EA-AD had Th2 dominant cytokines (IL-4 and IL-13), but Asian AD additionally activated robust Th17 and Th22 related cytokines (IL-17, IL-19, IL-22). The overexpression of the Th17 axis cytokines manifested lichenification with prominent epidermal hyperplasia. In addition, IgE was elevated in both phenotypes of AD, which was usually a feature distinct from PSO [23,24,75]. Genetically, 10 to 30% of the Asian population bear filaggrin mutations, and specific hotspots commonly reported in Asian AD were R501X and E2422X [76].

### 6.3. Coexisting AD and PSO

Psoriasis dermatitis (PD) means PSO and AD lesions appeared on the same person. Different names, including “PSO-AD overlapping syndrome”, “PSO and AD concomitant disease”, “PSO-Eczema”, or “eczema in psoriatico” have been used to describe this situation. The prevalence of PD ranges from 0.2 to 16.7%, depending on the definition and methods [10,12,13,77,78]. In consecutive cases, the onset of AD was generally earlier than PSO [12]. According to the retrospective study from a medical center, which included 1390 patients with PSO and another 30 patients having both PSO and AD, hand involvement rate was high (63%) and recalcitrant to treatment. Therefore, in a patient with a PSO history and refractory hand lesions, AD should be taken into consideration. In addition, more than one biologic agent was needed concomitantly in 22% of PD patients, and more than one consecutive biologic agent was required in 30% of them, which revealed the recalcitrant nature and disease complexity [10].

The prevalence rate of PD in children ranges from 1.5 to 3.7% [11,79]. A comparative study showed that characteristics of pediatric PD were much closer to PSO clinically and pathologically. Children with PD are often featured as pediatric PSO with flexural eczema [66]. In terms of location, facial involvement was higher, and scalp involvement was lower in PD compared to PSO. Nail changes were not significantly different between PD and PSO [79]. Moreover, IL-17 concentration was found to be significantly higher in PD than in AD or PSO. The role of the Th17 axis in PD was accordingly emphasized [9].

Interestingly, in patients with concurrent typical lesions of both PSO and AD, the two lesions can be elicited by their respective triggering factors, mechanical trauma (Koebner’s phenomenon) for PSO, and major house-dust mite allergens (atopy patch test) for AD [80].

### 6.4. Development of AD-like Dermatitis during PSO or AD Treatment

Paradoxical AD has been reported in patients treated with biologic agents for PSO and AD. Biologics related to paradoxical eczematous reactions have been observed in all categories: tumor necrosis factor-alpha inhibitor (TNF-ai), IL-12/23i, IL-17i, IL-23i, and IL-4/13i, but there is some difference among them [81]. A broad spectrum of clinical features, including facial eczema, dyshidrotic eczema, or generalized AD-like dermatitis, were recorded during biologics treatment for PSO (TNF-ai, IL-12/23i, IL-17i, IL-23i), while most commonly presented signs of dupilumab (IL-4/13i) were localized to the face and neck, so-called paradoxical face and neck erythema [81,82,83].

TNF-ai accounted for the most cases of eczematous reaction, and these cases have been discussed as tumor necrosis factor-alpha inhibitor-associated dermatitis [81,84]. Strong expression of IL-36, beta-defensin 2, Th2 cells, and Th17 cells were observed in the biopsy specimen from the PSO lesions of these cases [85]. It was suggested that the presence of eosinophils and plasma cells were the pathological features of tumor necrosis factor-α inhibitor-associated dermatitis [86], but eosinophils and plasma cells can also be seen in PSO. In contrast, IL-23i was rarely implicated in paradoxical eczematous reactions. Although this could be partially explained by the latest launch of IL-23i, data implied that IL-23i might have a lesser probability of inducing paradoxical eczema than IL-17i [81,84]. The onset time of eczema after starting biologics ranged widely, but mostly within 6 months [81].

Although the mechanisms remain unclear, several hypotheses have been proposed. First, the imbalance of the cytokine milieu is aggravated. For example, the inhibition of TNF-α by TNF-ai leads to uncontrolled plasmacytoid dendritic cells producing surplus IFN-α. Overproduction of IFN-α induces paradoxical skin reactions. Second, T cell polarization skewing drives paradoxical inflammation. For instance, inhibition of Th17 may direct Th2 polarization, causing paradoxical eczematous lesions. Third, genetic susceptibility might play a part. The polymorphism of AD genes might explain why the same biologic agent results in resolution in one person but triggers paradoxical AD in another [81,84,87].

Despite good efficacy elsewhere, treatment with dupilumab, an IL-4 and IL-13 receptor alpha antagonist for AD, induced a special form of facial dermatitis. Well-demarcated erythematous patches presented without prominent scales compared with typical eczema. Seldom itch or stinging sensation was accompanied, which was distinct from patients’ pre-existing AD. Histologically, psoriasiform dermatitis was found, but spongiosis, which was the main change of AD, was absent. Besides, this special drug-induced facial dermatitis showed no response to systemic itraconazole, oral and topical corticosteroids, topical calcineurin inhibitors, and emollients [82,83]. Although the incidence rate of paradoxical reaction was not low (estimated approximately 19%), most patients kept dupilumab without switch or discontinuation [81]. The pathophysiology proposed in one report was that the inhibition of the Th2 pathway by dupilumab might promote *Demodex* proliferation and increase IL-17-mediated inflammation [88].

### 6.5. Development of PSO during AD Treatment

A systematic review included 26 studies and 47 patients who developed PSO during treatment of dupilumab for AD, asthma, or alopecia areata (mainly AD: 43/47). None of the patients were children or adolescents (age range from 24 to 92 years old). The onset time after dupilumab initiation was 3.7 months. Discontinuation of dupilumab due to PSO accounted for 48% of patients. Complete resolution (40%) or improvement (48%) after discontinuation or giving PSO treatment were reported [89].

However, another retrospective study focused on children (≤ 18 years of age, *n* = 6) reported that the median onset time of psoriasiform dermatitis was eight months after dupilumab started. Besides, most of their psoriasiform lesions were ameliorated under medium-to-potent topical corticosteroid treatment [90]. Moreover, not only plaque-type PSO, but also pustular or reverse-form PSO induced by dupilumab was identified in case reports [91,92].

The suggested mechanism was that the inhibition of IL-4 by dupilumab activates Th1 and Th17 cells because IL-4 negatively regulates Th1 and Th17 cells, both of which are involved in PSO. Hitherto, no JAK inhibitor-induced PSO during AD treatment was identified in the literature [89,90].

It is unknown whether PSO induced by biologics for AD is unique to dupilumab since, during other biologics treatments for AD, such as tralokinumab and lebrikizumab (IL-13i), there have been no reports about the onset of PSO. More clinical experience and real-world data will be needed to answer this question.

### 6.6. Management

There has been no consensus on the optimal treatment of coexisting AD and PSO, but most conventional systemic therapies, including immunosuppressants (methotrexate, azathioprine, and cyclosporine) and phototherapy can be used for both diseases. Even for the target-specific DMARDS, namely Jak inhibitors and PDE4 inhibitors, they can be safely and effectively used for both conditions. In fact, biologic agents targeting one specific T-cell line appear to be non-beneficial for co-occurring diseases. Conversely, general or rather non-specific T-cell suppression shows efficacy [80] (Table 2).

On the other hand, concerning the cases of transformation from AD to PSO or vice versa during biologics treatment, discontinuation of the induced biologic agents and switching to a different mode of action is a generally accepted treatment strategy because transformation or paradoxical reaction is regarded as a class effect, not merely a drug-specific effect [87,89].

## 7. Conclusions

Overlapping manifestations and phenotypes between PSO and AD have been increasingly reported. Shared immunopathogenesis is in accordance with clinical findings. PSO and AD may be regarded as a spectrum of disease, rather than as a dichotomy. Since the diversity of skin features, disease course, onset time, and readjustment of cytokine milieu by medications, overlapping PSO and AD could be subdivided into five subtypes.

The sequence of disease onset varies from one another, but most of the patients experienced these diseases at different stages of life, according to other authors′ descriptions in the previous literature. For instance, patients developed AD in childhood, then enjoyed years of remission, and developed psoriasis later in adulthood. The concurrence or flare-up of both diseases at the same time or coexisting at the same period of life (e.g., when one flares up, the other subsides, or vice versa) was relatively rare [33,46,47].

The exploration of PSO-AD overlap condition is still in its early stage and largely confined to clinical manifestations. Cytokine markers or genetic analysis might provide a better understanding of the immune and barrier profile. Further studies on the molecular level could bring new disease classification and tailored treatment suggestions.

## Figures and Tables

**Table 1:** Comorbidities of autoimmune diseases between atopic dermatitis and psoriasis.

Comorbidities		Atopic Dermatitis	Psoriasis
**Gastroenterology**	Ulcerative colitis	V	Inconsistent data
	Crohn’s disease	V	V
	Celiac disease	V	V
**Dermatology**	Vitiligo	V	V
	Alopecia areata	V	V
**Atopy**	Allergic rhinitis	V	V
	Asthma	V	V
**Musculoskeletal** **disease**	Systemic lupus erythematosus	V	V
	Rheumatoid arthritis	V	V
**Autoimmune bullous disease**	Bullous pemphigoid	V	V
	Pemphigus	Unknown	V

**Table 2:** Reasonable choices for overlapping diseases.

	Typical PSO	Typical AD	AD-PSO Overlapping
**Treatment**			
**Topical agents**	Corticosteroids	Corticosteroids	Corticosteroids
	Vitamin D3 analog	Calcineurin inhibitor	Calcineurin inhibitor
	Retinoids	PDE4 inhibitor (crisaborole) JAK inhibitor (ruxolitinib)	
	Tar		
	Calcineurin inhibitor *		
**Conventional oral medications**	Methotrexate Acitretin Cyclosporin	Methotrexate * Azathioprine * Cyclosporin	Methotrexate Cyclosporin
**Phototherapy**	NBUVB ^#^	NBUVB ^#^	NBUVB ^#^
**Biologics**	IL-12/23i, IL-17i, IL-23i, TNF-αi	IL-4/13i, IL-13i	IL-12/23i ^?^
**Systemic small** **molecular drugs**	PDE4 inhibitor (apremilast) JAK inhibitor (upadacitinib) **	JAK inhibitor (baricitinib, upadacitinib, abrocitinib)	JAK inhibitor (upadacitinib) **

* Not licensed, but generally listed in worldwide treatment guidelines. ^#^ NBUVB: narrowband ultraviolet B. ^?^ Well-tolerated but without efficacy in atopic dermatitis [93,94]. ** Licensed for psoriatic arthritis.

## Data Availability

Not applicable.
